# Antiretroviral Drug Exposure and Response in Obese and Morbidly Obese People With Human Immunodeficiency Virus (HIV): A Study Combining Modelling and Swiss HIV Cohort Data

**DOI:** 10.1093/cid/ciad495

**Published:** 2023-08-21

**Authors:** Mattia Berton, Sara Bettonte, Felix Stader, Laurent Decosterd, Philip E Tarr, Françoise Livio, Matthias Cavassini, Dominique L Braun, Katharina Kusejko, Anna Hachfeld, Enos Bernasconi, Alexandra Calmy, Patrick Schmid, Manuel Battegay, Catia Marzolini, the Swiss HIV Cohort Study, Irene Abela, Karoline Aebi-Popp, Alexia Anagnostopoulos, Manuel Battegay, Enos Bernasconi, Dominique Laurent Braun, Heiner Bucher, Alexandra Calmy, Matthias Cavassini, Angela Ciuffi, Günter Dollenmaier, Matthias Egger, Luigia Elzi, Jan Fehr, Jacques Fellay, Hansjakob Furrer, Christoph Fux, Huldrych Günthard, Anna Hachfeld, David Haerry, Barbara Hasse, Hans Hirsch, Matthias Hoffmann, Irene Hösli, Michael Huber, David Jackson-Perry, Christian Kahlert, Laurent Kaiser, Olivia Keiser, Thomas Klimkait, Roger Dimitri Kouyos, Helen Kovari, Katharina Kusejko, Niklaus Labhardt, Karoline Leuzinger, Begona Martinez de Tejada, Catia Marzolini, Karin J Metzner, Nicolas Müller, Johannes Nemeth, Dunja Nicca, Julia Notter, Paolo Paioni, Giuseppe Pantaleo, Matthieu Perreau, Andri Rauch, Luisa Salazar-Vizcaya, Patrick Schmid, Roberto Speck, Marcel Stöckle, Philip Tarr, Alexandra Trkola, Gilles Wandeler, Maja Weisser, Sabine Yerly

**Affiliations:** Division of Infectious Diseases and Hospital Epidemiology, Departments of Medicine and Clinical Research, University Hospital Basel, Basel, Switzerland; Faculty of Medicine, University of Basel, Basel, Switzerland; Division of Infectious Diseases and Hospital Epidemiology, Departments of Medicine and Clinical Research, University Hospital Basel, Basel, Switzerland; Faculty of Medicine, University of Basel, Basel, Switzerland; Certara UK Limited, Sheffield, United Kingdom; Service and Laboratory of Clinical Pharmacology, Department of Laboratory Medicine and Pathology, University Hospital Lausanne and University of Lausanne, Lausanne, Switzerland; Kantonsspital Baselland, University of Basel, Bruderholz, Switzerland; Service and Laboratory of Clinical Pharmacology, Department of Laboratory Medicine and Pathology, University Hospital Lausanne and University of Lausanne, Lausanne, Switzerland; Service of Infectious Diseases, Lausanne University Hospital, University of Lausanne, Lausanne, Switzerland; Department of Infectious Diseases and Hospital Epidemiology, University Hospital Zurich, University of Zurich, Zurich, Switzerland; Department of Infectious Diseases and Hospital Epidemiology, University Hospital Zurich, University of Zurich, Zurich, Switzerland; Department of Infectious Diseases, University Hospital Bern, University of Bern, Bern, Switzerland; Division of Infectious Diseases, Ente Ospedaliero Cantonale Lugano, University of Geneva and University of Southern Switzerland, Lugano, Switzerland; Division of Infectious Diseases, University Hospital Geneva, University of Geneva, Geneva, Switzerland; Department of Infectious Diseases and Hospital Epidemiology, Cantonal Hospital St Gallen, St Gallen, Switzerland; Division of Infectious Diseases and Hospital Epidemiology, Departments of Medicine and Clinical Research, University Hospital Basel, Basel, Switzerland; Faculty of Medicine, University of Basel, Basel, Switzerland; Division of Infectious Diseases and Hospital Epidemiology, Departments of Medicine and Clinical Research, University Hospital Basel, Basel, Switzerland; Faculty of Medicine, University of Basel, Basel, Switzerland; Service and Laboratory of Clinical Pharmacology, Department of Laboratory Medicine and Pathology, University Hospital Lausanne and University of Lausanne, Lausanne, Switzerland; Department of Molecular and Clinical Pharmacology, University of Liverpool, Liverpool, United Kingdom

**Keywords:** obesity, antiretrovirals, drug exposure, drug response, HIV

## Abstract

**Background:**

Obesity is increasingly prevalent among people with HIV (PWH) and can possibly result in suboptimal antiretroviral drug (ARV) exposure and response. However, this has not been thoroughly evaluated given that obese PWH are underrepresented in clinical trials. We performed virtual trials using physiologically based pharmacokinetic (PBPK) modelling combined with observed clinical data to provide ARV dosing guidance in obese individuals.

**Methods:**

Each trial included a cohort of virtual adults with a body mass index (BMI) between 18.5 and 60 kg/m^2^. Therapeutic drug-monitoring data from the Swiss HIV Cohort Study (SHCS) were used to verify the predictive performance of the model. Subsequently, the model was applied to predict the pharmacokinetics of ARVs for different obesity classes. The association between ARV plasma concentrations and virological response was investigated in obese and nonobese individuals.

**Results:**

The PBPK model predicted an average reduction in ARV exposure of ∼20% and trough concentrations of ∼6% in obese (BMI ≥30 kg/m^2^) compared with nonobese (BMI: 18.5–25 kg/m^2^) individuals, consistent with observed clinical data. Etravirine and rilpivirine were the most impacted, especially in individuals with BMI >40 kg/m^2^ whose trough concentrations were below the clinical target threshold. Obese PWH in the SHCS did not have a higher rate of unsuppressed viral load than nonobese PWH.

**Conclusions:**

The concentrations of ARVs are modestly reduced in obese individuals, with no negative impact on the virological response. Our data provide reassurance that standard doses of ARVs are suitable in obese PWH, including those who gained substantial weight with some of the first-line ARVs.

## Teaser

Antiretroviral dose adjustment is not required a priori in obese people with HIV (PWH). Despite a 20% reduction in antiretroviral exposure and trough concentrations below target thresholds for etravirine and rilpivirine, virological response was similar between obese and nonobese PWH.

Obesity represents one of the biggest challenges that health systems face due to its increasing prevalence, associated comorbidities, and increased mortality. According to World Health Organization estimates, in 2016, 39% and 13% of the worldwide population was overweight (body mass index [BMI]: 25–30 kg/m^2^) and obese (BMI: ≥30 kg/m^2^), respectively [1–3]. Thanks to effective antiretroviral drugs (ARVs) and the related improvement in health, people with human immunodeficiency virus (HIV; PWH) are part of this epidemic and are affected by obesity at a rate similar to the general population [2]. Obesity does not only represent a health risk but leads to physiological changes that can reduce drug exposure, possibly resulting in loss of effectiveness [4, 5]. However, this question has not been thoroughly evaluated, in part because obese PWH are underrepresented in clinical trials.

Physiologically based pharmacokinetic (PBPK) modelling is a mathematical tool, recognised by regulatory bodies, that combines drug properties, human physiology, and clinical observed data to simulate virtual clinical trials of interest. PBPK modelling can be applied to study the pharmacokinetics of drugs in special populations such as the elderly [6], children [7], pregnant women [8], and obese individuals [5]. However, to date, it has not been used to fully investigate the impact of obesity on the pharmacokinetics of ARVs, which represents an important knowledge gap considering that several first-line ARVs have been associated with weight gain (increase in weight: 2 kg at 48 wk after initiating an integrase inhibitor; 1.7 kg over 18 mo after switching from tenofovir disoproxil fumarate [TDF] to tenofovir alafenamide) [3, 9, 10].

This study aimed to perform virtual trials using PBPK modelling combined with therapeutic drug monitoring (TDM) data and the corresponding viral load data obtained from PWH enrolled in the Swiss HIV Cohort Study (SHCS) to determine the exposure and response to ARVs in obese and nonobese PWH and provide dosing guidance.

## METHODS

We took several steps to analyse the impact of obesity on the ARVs exposure and response. First, we implemented our in-house PBPK model with our recently published virtual White obese population [4] to simulate the pharmacokinetics of ARVs in nonobese and obese individuals. Next, we verified the simulations against the TDM data collected during the follow-up visits of the SHCS (multiple drug levels per individual were allowed [more information on the SHCS in the homonymous section in the Supplementary Material]). We then applied the verified model to extrapolate the pharmacokinetics across different obesity classes. Finally, the clinical relevance of obesity-related changes on drug response was evaluated by analyzing HIV viral load and the corresponding TDM values in obese and nonobese individuals in the SHCS.

### PBPK Model and Virtual Obese Population

Our previous PBPK model developed in Matlab 2020a [11] was implemented with mathematical functions describing anatomical, physiological, and biological changes occurring in a White obese population aged 20–50 years and a BMI ranging from 18.5 to 60 kg/m^2^ [4]. The model performance to predict the pharmacokinetics of non-HIV drugs in obese individuals has been demonstrated previously [5].

### HIV Drug Model Development and Verification With Data of the SHCS

All ARV drug models, except for doravirine (Supplementary Table 1), were previously developed and verified to predict the pharmacokinetics in healthy and elderly PWH [6, 12]. For the present study, the predictive performance of the drug models in obese individuals was verified using TDM data of ARVs (ie, ritonavir, darunavir/ritonavir, efavirenz, etravirine, rilpivirine, doravirine, dolutegravir, bictegravir, raltegravir, emtricitabine, and TDF). The TDM data were excluded if the participant’s age was younger than 20 or older than 50 years to avoid the age-related confounding effect on the pharmacokinetics, if a nonstandard ARV dosage was used, and if concurrent medications with inhibitory or inducing effects were used. The pharmacokinetic parameters including peak plasma concentration (C_max_), area under the curve to time *t* (AUC_t_), and trough concentration (C_τ_) were derived from the TDM data using noncompartmental analysis.

A literature search was also performed to identify pharmacokinetic studies with ARVs conducted in obese and nonobese individuals. Data on the study design, characteristics of the participants, and pharmacokinetic results were collected. When multiple studies were found, the weighted mean and standard deviation or geometric mean and coefficient of variance were reported. Available concentration-time profiles were digitalized using GetData Graph Digitizer V.2.26. The virtual trials were conducted by matching the participants’ demographics (eg, age, proportion of females, and BMI) and the ARV dosing regimen (Supplementary Table 2). The models were considered verified if the simulations were within 2-fold of observed data.

### Analysis of Antiretroviral Drug Exposure Across BMI Categories

The PBPK model implemented with continuous functions describing the physiology in obese individuals up to a BMI of 60 kg/m^2^ allowed us to conduct virtual trials for people in the different obesity classes, including BMI 30–35, 35–40, 40–50 and 50–60 kg/m^2^. The C_max_, AUC_t_, and C_τ_ were normalized to those obtained from the virtual trial in lean individuals (BMI: 18.5–25 kg/m^2^) to derive the effect of obesity expressed as fold-change.

### Drug Response in Obese Versus Nonobese Participants in the SHCS

The viral load was analyzed in conjunction with the TDM data to evaluate whether a decrease in ARV exposure in obese PWH is associated with a viral load of more than 50 copies/mL (the viral load measured on the date of the TDM assessment was considered for this evaluation). Specifically, we calculated the percentage of PWH with concentrations below the clinical target threshold reported in the literature (except for emtricitabine and tenofovir as no plasma concentration effect has been established for these drugs) and, among those, the percentage of PWH with a viral load greater than 50 copies/mL.

## RESULTS

### Clinical Data From the SHCS

Rich TDM datasets were obtained from the SHCS database for all evaluated ARVs apart from etravirine, rilpivirine, doravirine, and tenofovir, for which only 10–20 data points were available from obese individuals. These TDM measurements showed that the AUC_t_ of most ARVs was reduced in obese compared with nonobese PWH (Table 1).

**Table 1.** Observed and Predicted Pharmacokinetic Parameters for Various Antiretrovirals in Nonobese and Obese Individuals

			Nonobese Individuals				Obese Individuals				Ratio Predicted/Observed		Ratio Obese/Nonobese		Ratio (Predicted Ratio Obese/Nonobese)/(Observed Ratio Obese/Nonobese)
	Parameter	Unit	Observed		Predicted		Observed		Predicted		Nonobese	Obese	Observed	Predicted	
			GM	CV%	GM	CV%	GM	CV%	GM	CV%					
Protease inhibitors															
** **Drug	BMI	kg/m^2^	19–30		19–30		30–51		30–51		…	…	1.65		…
** **Ritonavir	C_max_	ng/mL	480	170.2	604	51.7	520	110.9	440	68.7	1.26	0.85	1.08	0.73	0.68
** **Dose	t1/2	h	5.4	124.5	6.3	23.3	6.3	104	7.5	46.3	1.17	1.19	1.17	1.18	1.01
** **100 mg QD	AUC_t_	ng*h/mL	5740	55	7006	83.6	4988	104	5665	115.6	1.22	1.14	0.87	0.81	0.93
	C_τ_	ng/mL	91.4	144.3	70.1	231.8	71.3	85.6	75.6	310	0.77	1.06	0.78	1.08	1.38
** **Drug	BMI	kg/m^2^	21–30		21–30		30–42		30–42		…	…	1.41		…
** **Darunavir/ritonavir	C_max_	ng/mL	6442	26	5979	46	4367	…	4990	37.2	0.93	1.14	0.68	0.83	1.22
** **Dose	t1/2	h	13.4	72.6	11.3	56.8	12.8	…	16.4	39.4	0.84	1.28	0.96	1.46	1.52
** **800/100 mg QD	AUC_t_	ng*h/mL	73 282	31	87 103	65.6	58 641	39	74 652	50.7	1.19	1.27	0.80	0.86	1.08
	C_τ_	ng/mL	1532	53.5	1626	120.5	1556	56.9	1653	79.1	1.06	1.06	1.02	1.02	1.00
Nonnucleoside reverse transcriptase inhibitors															
** **Drug	BMI	kg/m^2^	19–30		19–30		30–60		30–60		…	…	1.84		…
** **Efavirenz	C_max_	ng/mL	3172	93.9	3847	29.7	2715	…	3097	26.5	1.21	1.14	0.86	0.81	0.94
** **Dose	t1/2	h	20.2	38.5	26.9	32.7	22.2	…	36.4	34.4	1.34	1.64	1.10	1.35	1.23
** **600 mg QD	AUC_t_	ng*h/mL	52 234	68.6	51 693	43.8	39 267	58.9	40 536	41	0.99	1.03	0.75	0.78	1.04
	C_τ_	ng/mL	1751	99.6	1654	55.3	1512	58.5	1389	49	0.94	0.92	0.86	0.84	0.98
** **Drug	BMI	kg/m^2^	20–31		20–31		30–51		30–51		…	…	1.59		…
** **Etravirine*	C_max_	ng/mL	949	298	882	348	534	195	685	238	0.93	…	…	0.78	…
** **Dose	t1/2	h	12.8	6.9	14.5	4.0	19.7	7.2	17.3	4.8	1.13	0.88	1.54	1.19	0.77
** **200 mg BID	AUC_t_	ng*h/mL	7970	2319	7436	3511	3289	1199	5494	2404	0.93	…	…	0.74	…
	C_τ_	ng/mL	464	157	467	267	313	252	340	186	1.01	1.09	0.67	0.73	1.09
** **Drug	BMI	kg/m^2^	22–30		22–30		30–38		30–38		…	…	1.31		…
** **Rilpivirine	C_max_	ng/mL	159.7	31	148.5	37.6	147.7	62.3	148.2	33.4	0.93	1.00	0.92	1.00	1.09
** **Dose	t1/2	h	47.2	23	30.9	7.5	…	…	40.4	8.3	0.65	…	…	1.31	…
** **25 mg QD	AUC_t_	ng*h/mL	2333	30.2	2457	761.5	1981	19.1	2444	672.8	1.05	1.23	0.85	0.99	1.16
	C_τ_	ng/mL	75.2	39	71.9	28.6	89.2	66.2	74.8	25.4	0.96	0.84	1.19	1.04	0.87
** **Drug	BMI	kg/m^2^	20–30		20–30		30–41		30–41		…	…	1.42		…
** **Doravirine	C_max_	ng/mL	1226	7.7	1353	21.6	…	…	1122	22.9	1.10	…	…	0.83	…
** **Dose	t1/2	h	13.5	33.8	14.0	22	…	…	17.7	17.4	1.03	…	…	1.27	…
** **100 mg QD	AUC_t_	ng*h/mL	17 498	7.5	17 550	22.8	…	…	15 103	23.7	1.00	…	…	0.86	…
	C_τ_	ng/mL	404	9.7	349	30.6	513	21.6	348	27.9	0.86	0.68	1.27	1.00	0.79
Integrase inhibitors															
** **Drug	BMI	kg/m^2^	19–30		19–30		30–42		30–42		…	…	1.47		…
** **Dolutegravir	C_max_	ng/mL	3317	50.9	3116	33.3	2884	41.2	2552	35.3	0.94	0.88	0.87	0.82	0.94
** **Dose	t1/2	h	12.6	42.3	11.8	60.9	…	…	15.4	57.6	0.94	…	…	1.31	…
** **50 mg QD	AUC_t_	ng*h/mL	45 446	57.1	44 038	57.1	43 415	47	39 922	54.8	0.97	0.92	0.96	0.91	0.95
	C_τ_	ng/mL	889	82.7	785	119.8	940	65.8	890	96.1	0.88	0.95	1.06	1.13	1.07
** **Drug	BMI	kg/m^2^	19–30		19–30		30–44		30–44		…	…	1.51		…
** **Bictegravir	C_max_	ng/mL	5238	35.2	4574	46.3	…	…	3767	46.2	0.87	…	…	0.82	…
** **Dose	t1/2	h	20.6	36	26.3	64.8	…	…	32.8	63.9	1.27	…	…	1.25	…
** **50 mg QD	AUC_t_	ng*h/mL	85 786	36	82 802	61.3	…	…	70 068	60.2	0.97	…	…	0.85	…
	C_τ_	ng/mL	2373	40.2	2392	86.6	…	…	2180	79.1	1.01	…	…	0.91	…
** **Drug	BMI	kg/m^2^	19–30		19–30		30–52		30–52		…	…	1.67		…
** **Raltegravir	C_max_	ng/mL	2405	52.8	2054	23.7	…	…	1551	25.5	0.85	…	…	0.75	…
** **Dose	t1/2	h	4.0	…	5.5	24.8	…	…	8.4	14.2	1.40	…	…	1.51	…
** **400 mg BID	AUC_t_	ng*h/mL	7722	51.9	7184	28.3	…	…	5425	27.4	0.93	…	…	0.76	…
	C_τ_	ng/mL	142	80.6	110	55.7	171	84.2	113	45.2	0.77	0.66	1.20	1.03	0.86
Nucleoside/nucleotide reverse transcriptase inhibitors															
** **Drug	BMI	kg/m^2^	21–33		21–33		…		30–46		…	…	1.41		…
** **Emtricitabine*	C_max_	ng/mL	1640.2	593.9	1630.4	136.1	…	…	1226.52	128.65	0.99	…	…	0.75	…
** **Dose	t1/2	h	9.4	2.8	17.4	4.5	…	…	23.34	3.33	1.86	…	…	1.34	…
** **200 mg QD	AUC_t_	ng*h/mL	9589.4	2720.1	10 535.9	289.7	…	…	7615.27	319.44	1.10	…	…	0.72	…
	C_τ_	ng/mL	67.1	35.6	90.6	14.3	…	…	72.21	8.46	1.35	…	…	0.80	…
** **Drug	BMI	kg/m^2^	20–30		20–30		36–46		36–46		…	…	…		…
** **Tenofovir*	C_max_	ng/mL	300.5	76.2	300.	46.0	263	79	219.58	27.14	1.00	0.83	…	…	…
** **Dose	t1/2	h	14.9	4	17.1	2.8	13	3	22.7	3.0	1.15	1.75	…	…	…
** **300 mg QD non-obese	AUC_t_	ng*h/mL	2858.4	727.5	3035.0	416.1	2346	643	2234.7	268.7	1.06	0.95	…	…	…
** **245 mg QD obese	C_τ_	ng/mL	58.2	18.6	64.8	13.1	47	15	52.9	8.67	1.11	1.13	…	…	…

All results are reported as GM and CV% unless otherwise indicated. *Values are mean and standard deviation.

Abbreviations: AUC_t_, area under the curve to time *t*; BID, twice daily; BMI, body mass index; C_max_, peak concentration; C_τ_, trough concentration; CV%, coefficient of variance; GM, geometric mean; QD, once daily; t_1/2_, elimination half-life.

### Comparison Between Observed Data and PBPK Model Predictions

#### Protease Inhibitors

**Figure 1.**
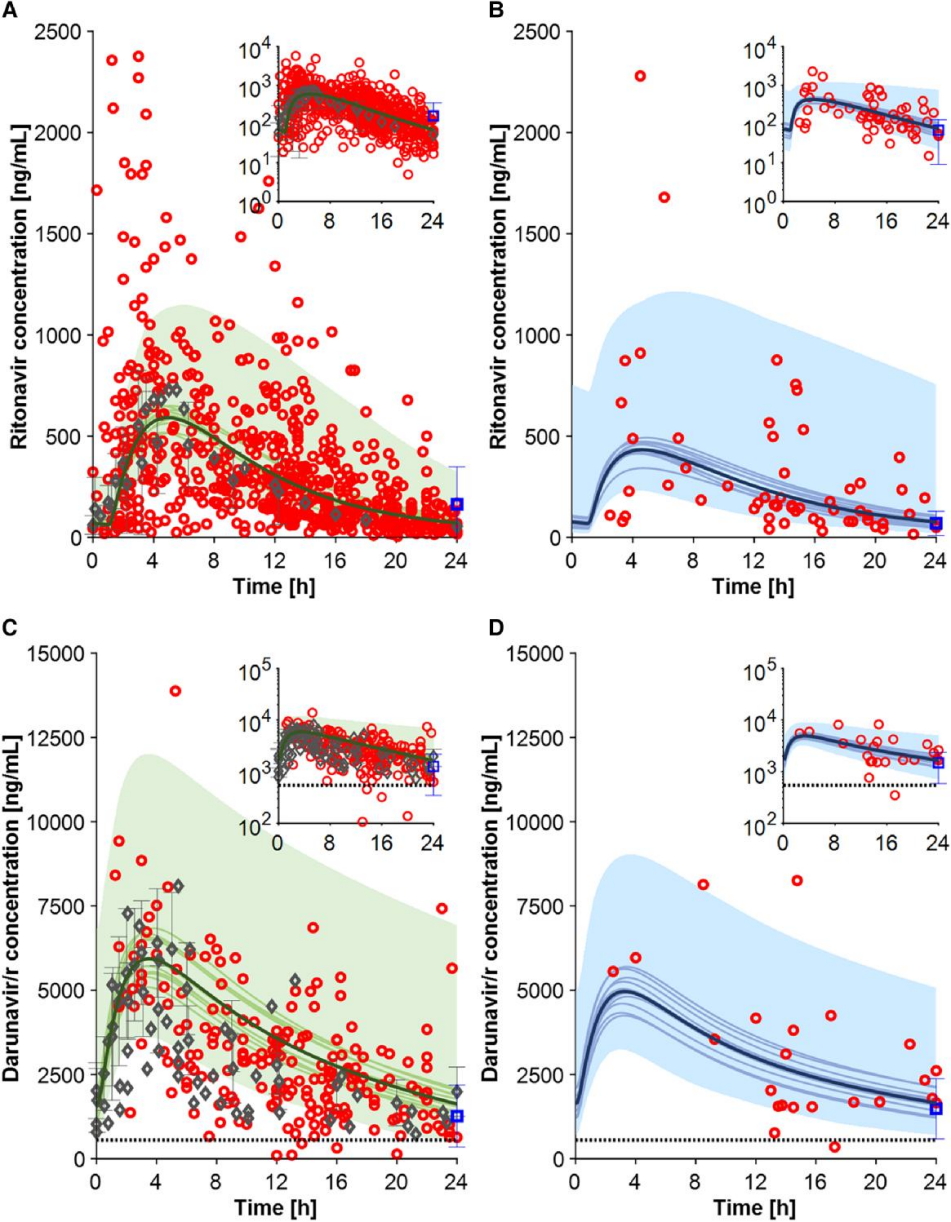
Full profile for ritonavir 100 mg QD in nonobese (*A*) and obese (*B*) individuals and for darunavir/ritonavir 800/100 QD in nonobese (*C*) and obese (*D*) individuals. Gray diamond markers represent the clinical observed data obtained from the literature, the blue square markers represent the C_τ_ value reported by Madelain et al [24], and the red circles represent the clinical data from the Swiss HIV Cohort Study. The simulation results conducted with 100 virtual individuals, specifically the mean of all virtual trials, the mean of each virtual trial, and the 90% normal range, are shown in the figures as solid bold lines, solid lines, and shaded areas, respectively. The dashed lines represent the clinical efficacy threshold. Abbreviations: Darunavir/r, darunavir/ritonavir; HIV, human immunodeficiency virus; QD, once daily.

The PBPK model correctly predicted the pharmacokinetics of the CYP3A4 substrates ritonavir (100 mg once daily [QD]) and darunavir/ritonavir (800/100 mg QD) in obese and nonobese individuals with predictions within 1.5-fold of observed clinical data (Table 1). Furthermore, most TDM data points were within the 90% range of predictions, indicating the model’s ability to describe the population variability in both populations (Figure 1). Ritonavir AUC_t_ was predicted to decrease by 19% for a population with a BMI of 30–51 kg/m^2^, in line with clinical data. The observed ritonavir C_τ_ decreased from 91.4 in nonobese to 71.3 ng/mL in obese individuals while the corresponding predicted values were 70.1 and 75.6 ng/mL. The model predicted a 14% decrease in darunavir/ritonavir AUC_t_ for a BMI of 30–42 kg/m^2^ while C_τ_ remained unchanged, in agreement with the observed data.

#### Nonnucleoside Reverse Transcriptase Inhibitors

**Figure 2.**
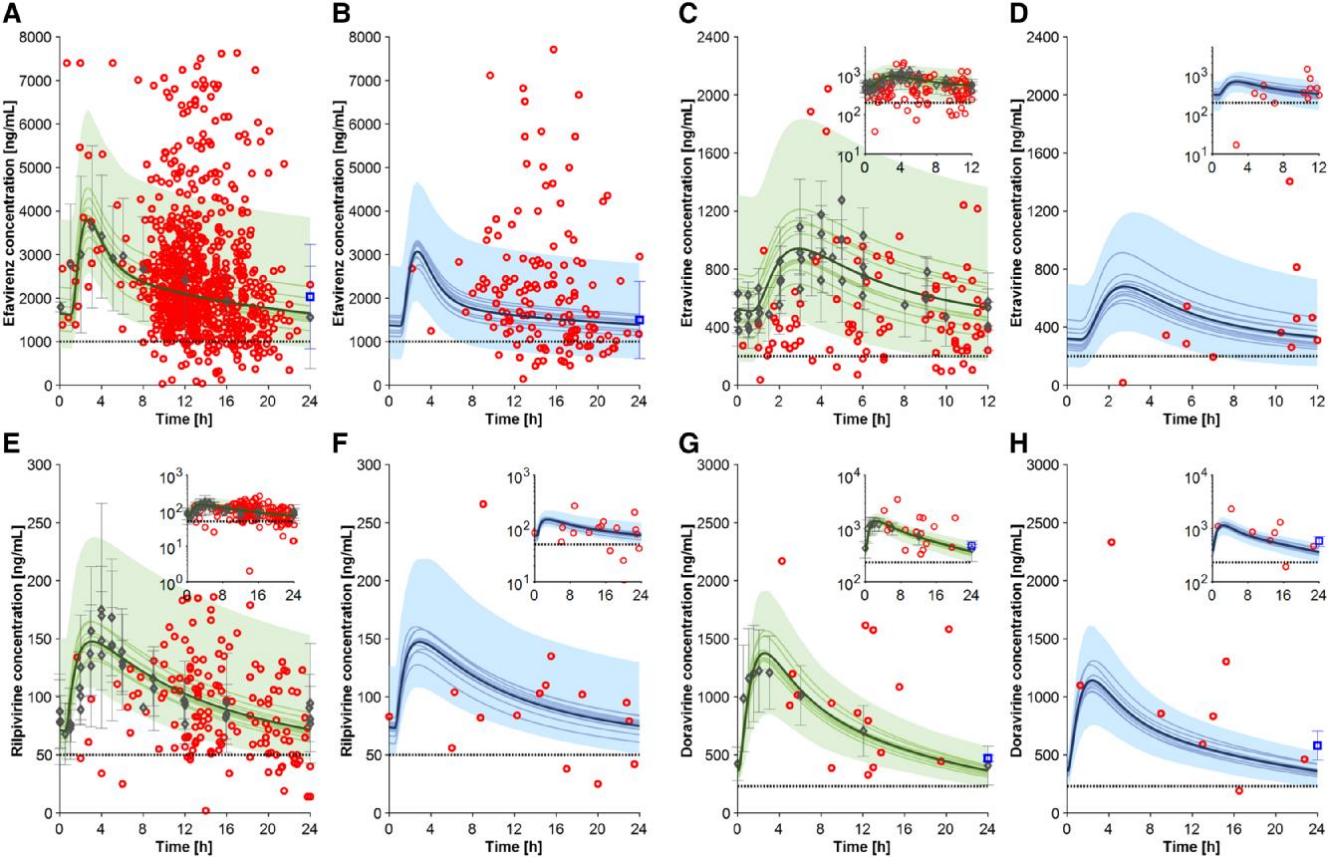
Full profile for efavirenz 600 mg QD in nonobese (*A*) and obese (*B*) individuals; etravirine 200 mg BID in nonobese (*C*) and obese (*D*) individuals; rilpivirine 25 mg QD in nonobese (*E*) and obese (*F*) individuals; and doravirine 100 mg QD in nonobese (*G*) and obese (*F*) individuals. Gray diamond markers represent the clinical observed data collected from the literature, the blue square marker for efavirenz represents the C_τ_ value reported by Madelain et al [24] and the one for doravirine the C_τ_ value reported by Zino [25], and the red circles illustrate the clinical data from the Swiss HIV Cohort Study. The simulation results conducted with 100 virtual individuals, specifically the mean of all virtual trials, the mean of each virtual trial, and the 90% normal range, are shown in the figures as solid bold lines, solid lines, and shaded areas, respectively. The dashed lines represent the clinical efficacy threshold. Abbreviations: BID, twice daily; HIV, human immunodeficiency virus; QD, once daily.

Efavirenz (600 mg QD), etravirine (200 mg twice daily [BID]), rilpivirine (25 mg QD), and doravirine (100 mg QD) simulations were, for the most part, within 1.25-fold of observed data (Table 1). The model was able to capture the variability in the nonobese and obese populations (Figure 2*C–H* ), except for efavirenz for which the observed variability was underpredicted (Figure 2*A* and 2*B* ). Possible explanations could relate to efavirenz absorption or the fact that CYP2B6 polymorphisms were not considered in the model. Efavirenz AUC_t_ and C_τ_ were predicted to be reduced by 22% and 16% when considering a BMI of 30–60 kg/m^2^, in line with the TDM data (Table 1). A comparable effect was observed for etravirine, whereas the predicted and observed rilpivirine and doravirine AUC_t_ and C_τ_ were minimally impacted.

#### Integrase Inhibitors

**Figure 3.**
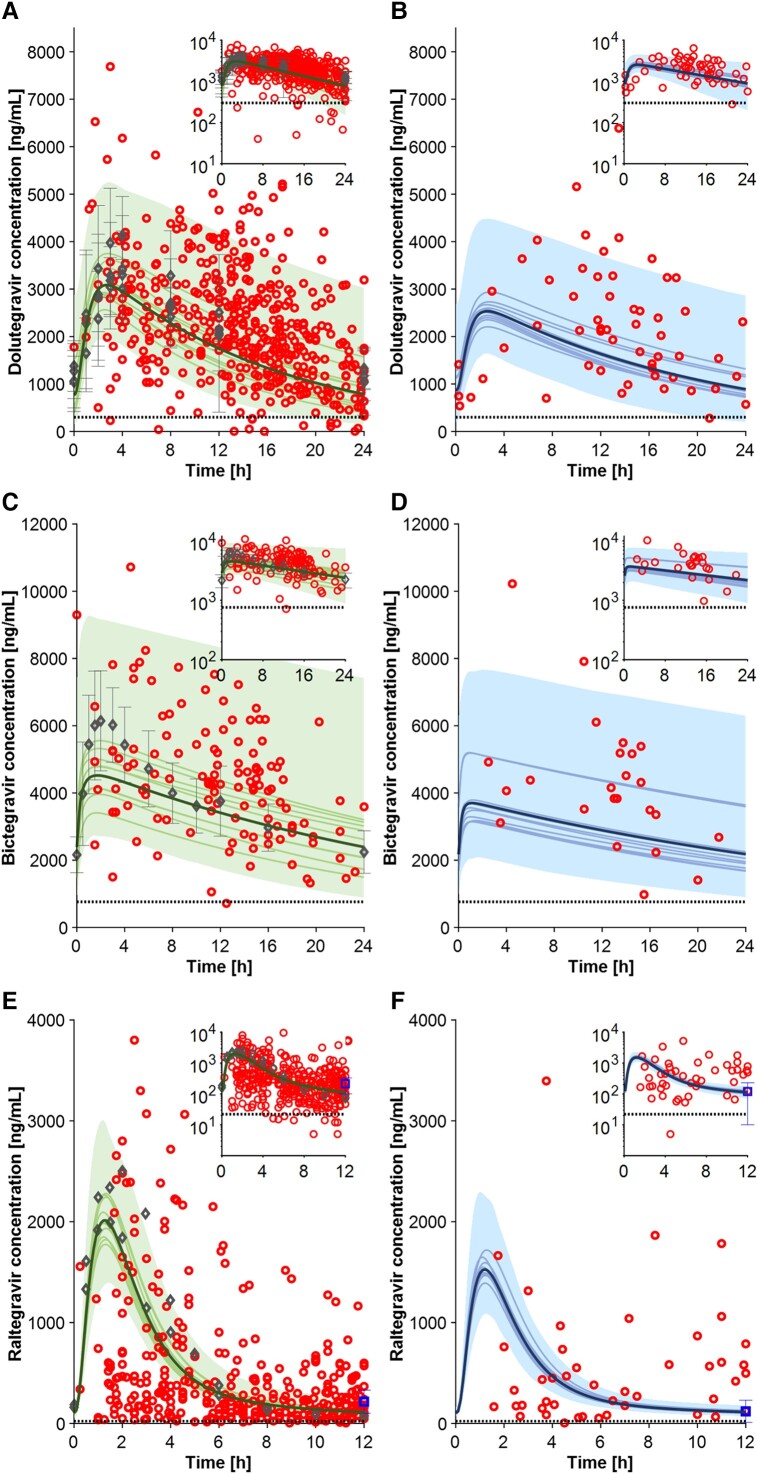
Full profile for dolutegravir 50 mg QD in nonobese (*A*) and obese (*B*) individuals; for bictegravir 50 mg QD in nonobese (*C*) and obese (*D*) individuals; and for raltegravir 400 mg BID in nonobese (*E*) and obese (*F*) individuals. Gray diamond markers depict the clinical observed data obtained from the literature, the blue square markers represent the C_τ_ value reported by Madelain et al [24], and the red circles illustrate the clinical data from the Swiss HIV Cohort Study. The simulation results conducted with 100 virtual individuals, specifically the mean of all virtual trials, the mean of each virtual trial, and the 90% normal range, are shown in the figures as solid bold lines, solid lines, and shaded areas, respectively. The dashed lines represent the clinical efficacy threshold. Abbreviations: BID, twice daily; HIV, human immunodeficiency virus; QD, once daily.

The model correctly predicted the pharmacokinetics of dolutegravir (50 mg QD), bictegravir (50 mg QD), and raltegravir (400 mg BID). For all 3 ARVs, the simulated pharmacokinetic parameters were within 1.5-fold of observed data in both studied groups. For bictegravir and raltegravir, due to the limited observed data points, no comparison could be made for obese and nonobese participants (Table 1). The population variability was well captured for dolutegravir (Figure 3*A* and 3*B* ) and bictegravir (Figure 3*C* and 3*D* ), whereas it was underpredicted for raltegravir (Figure 3*E* and 3*F* ), as also observed previously for other populations [6]. Obesity was predicted to minimally impact dolutegravir AUC_t_ and C_τ_, consistent with the observed data when considering a BMI of 30–42 kg/m^2^.

#### Nucleoside/Nucleotide Reverse Transcriptase Inhibitors

**Figure 4.**
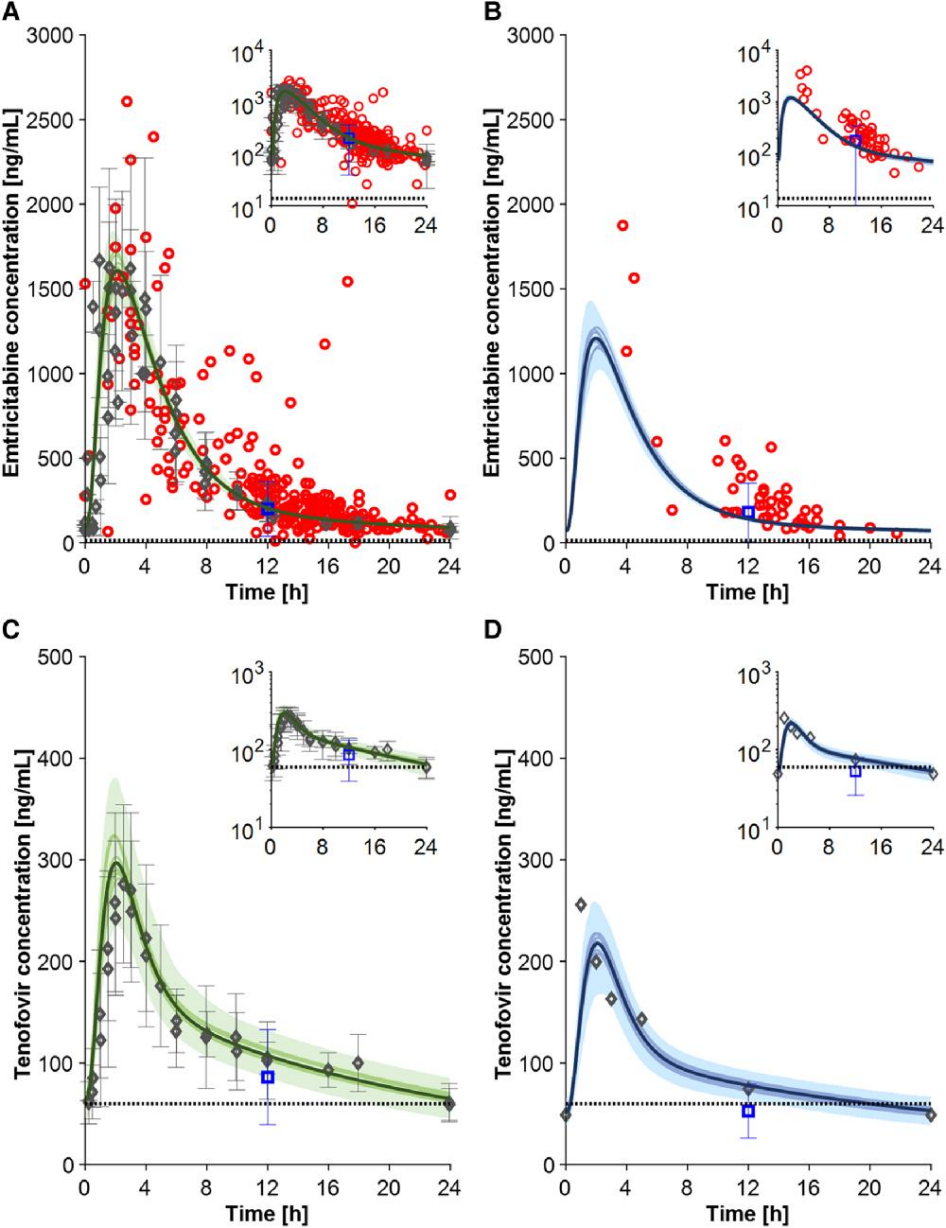
Full profile for emtricitabine 200 mg QD in nonobese (*A*) and obese (*B*) individuals and for tenofovir 300 mg in nonobese (*C*) and obese (*D*) individuals. Gray diamond markers depict the clinical observed data obtained from the literature, the blue square markers represent the C_τ_ value reported by Madelain et al [24] and Muzard et al [29], and the red circles illustrate the clinical data from the Swiss HIV Cohort Study. The simulation results conducted with 100 virtual individuals, specifically the mean of all virtual trials, the mean of each virtual trial, and the 90% normal range, are shown in the figures as solid bold lines, solid lines, and shaded areas, respectively. Abbreviations: HIV, human immunodeficiency virus; QD, once daily.

The PBPK model predicted well the pharmacokinetics of the renally eliminated drugs emtricitabine (200 mg QD) and TDF (300 mg QD). For emtricitabine, the mean predictions were in agreement with the mean observed data (Table 1); however, the population variability was not fully captured by the model (Figure 4*A* and 4*B* ). For tenofovir, the visual comparison of the observed and simulated concentration-time profiles was deemed good, and it was further confirmed by the very small fold error calculated for all pharmacokinetic parameters (Figure 4*C* and 4*D* ).

### Pharmacokinetic Changes of Antiretrovirals Across Obesity Classes and Clinical Relevance

**Figure 5.**
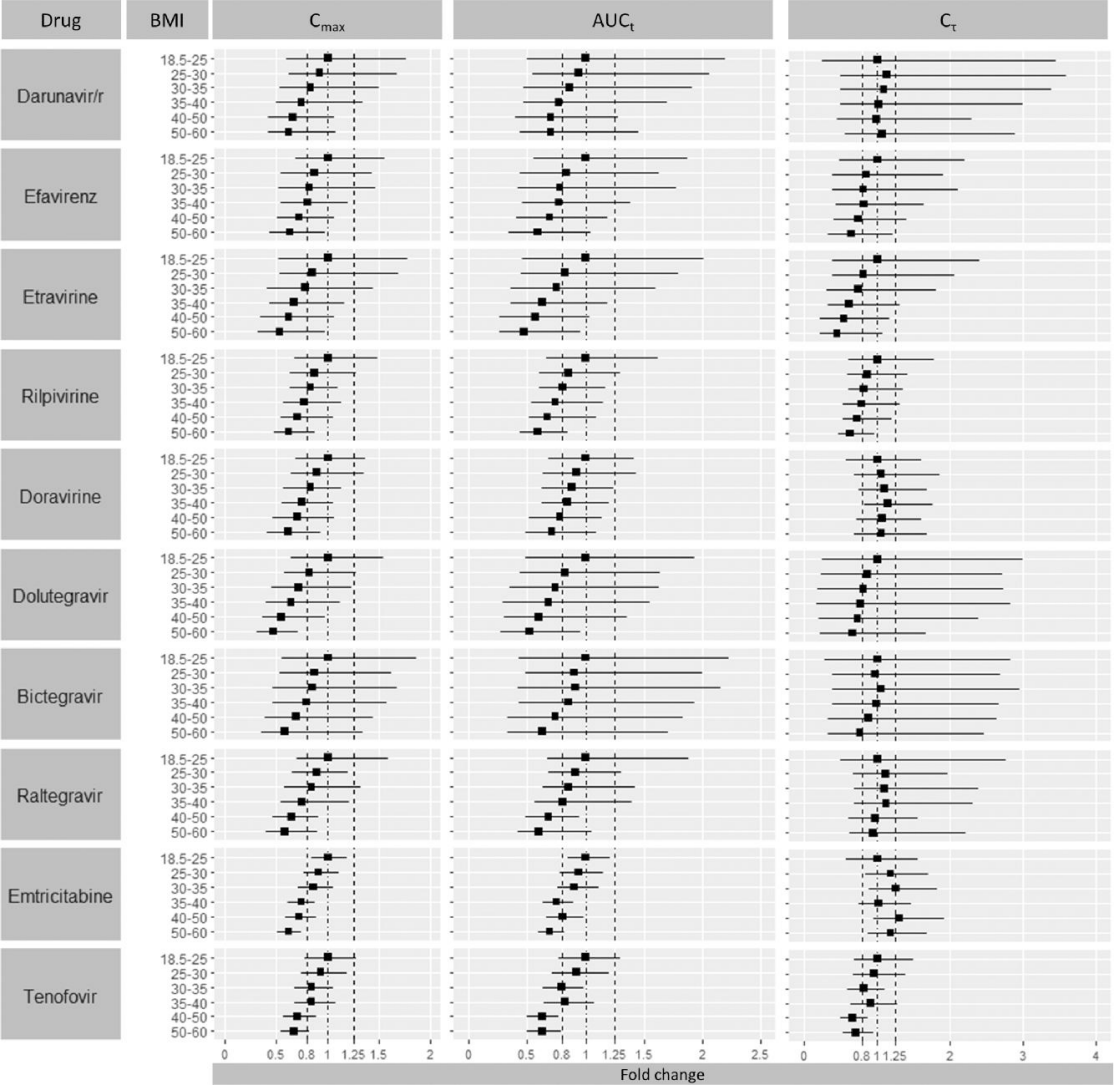
Predicted pharmacokinetic parameter fold-changes across different BMI categories. Data are expressed as geometric mean and 5th and 95th percentiles. Abbreviations: AUC_t_, area under the curve to time *t*, BMI, body mass index, Cmax, peak concentration, C_τ_, trough concentration.

The effect of obesity on the pharmacokinetics of the evaluated ARVs was investigated in 6 virtual clinical trials, each focusing on a different BMI category (Figure 5 and Supplementary Table 3). Furthermore, the clinical relevance of the pharmacokinetic change was evaluated by calculating the percentage of virtual individuals below the clinical efficacy target threshold of the ARV of interest (Table 2) and by comparing the proportion of obese versus nonobese individuals with a viral load greater than 50 copies/mL (Table 3).

**Table 2.** Percentage of Virtual Individuals Across Different BMI Categories With Predicted Plasma Concentrations Below the Trough Efficacy Target Threshold

	Darunavir/Ritonavir	Efavirenz	Etravirine	Rilpivirine	Doravirine	Dolutegravir	Bictegravir	Raltegravir
Target threshold (ng/mL)	550 [13]	700 [14]	300 [15]	50 [16]	230 [17]	300 [18]	760 [19]	20 [20]
BMI: 18.5–25 kg/m^2^	10	1	21	4	19	9	8	0
BMI: 25–30 kg/m^2^	0	4	41	6	18	9	3	0
BMI: 30–35 kg/m^2^	0	3	38	4	8	15	3	0
BMI: 35–40 kg/m^2^	3	3	54	11	2	15	4	0
BMI: 40–50 kg/m^2^	1	4	58	24	12	14	7	0
BMI: 50–60 kg/m^2^	0	9	72	45	11	13	6	0

Abbreviation: BMI, body mass index.

**Table 3.** Percentage of Participants of the Swiss HIV Cohort Study With Antiretroviral Drug Concentrations Below the Efficacy Target Threshold and With Viral Load Above 50 Copies/mL Categorized by BMI

Antiretroviral Drug	Darunavir/Ritonavir		Efavirenz		Etravirine		Rilpivirine		Doravirine		Dolutegravir		Bictegravir		Raltegravir	
BMI categories, kg/m^2^	18.5–30	>30	18.5–30	>30	18.5–30	>30	18.5–30	>30	18.5–30	>30	18.5–30	>30	18.5–30	>30	18.5–30	>30
Number of patients	322	38	1867	96	84	10	107	9	10	4	340	39	65	15	379	43
Proportion female, %	25.8	47.4	26.7	45.8	31	90	23.4	33.3	50	100	21.5	48.7	33.8	93.3	26.6	27.9
Mean age, y	39.3	39.4	38.7	41	39	42.9	39.4	45.8	38.4	43	38.9	43.4	39.1	42.7	41	44.7
Mean BMI, kg/m^2^	23.8	33.6	23.2	33.6	24	32.8	24.1	32.4	24.3	36.4	23.6	33.4	24.6	33.7	23.7	35.2
% PWH with concentration below efficacy threshold	4.3	0	2.3	5.2	32.1	10	15	0	0	25	2.1	2.6	1.5	0	4	4.7
% PWH with virological failure amongst those with concentration below efficacy threshold	1.6	0	1.1	1.1	11.9	0	1.9	0	0	0	0.3	0	0	0	1.6	0

Abbreviations: BMI, body mass index; HIV, human immunodeficiency virus; PWH, people with HIV.

#### Protease Inhibitors

Obesity was predicted to decrease darunavir/ritonavir AUC_t_ and C_max_ by more than 25%—hence, below the Food and Drug Administration bioequivalence limit (ie, 0.8)—in individuals with a BMI >40 kg/m^2^ (Figure 5). However, the C_τ_ levels were predicted to be unaltered across all obesity classes, which was further supported by the small number of individuals with predicted darunavir/ritonavir C_τ_ levels below the target threshold (Table 2). The non–clinically significant effect of obesity on darunavir/ritonavir exposure was also consistent with the SHCS data showing that obese individuals receiving darunavir/ritonavir treatment did not have a higher rate of viral load greater than 50 copies/mL due to reduced exposure compared with nonobese individuals (Table 3).

#### Nonnucleoside Reverse Transcriptase Inhibitors

The AUC_t_ of nonnucleoside reverse transcriptase inhibitors was also predicted to be reduced with increasing degrees of obesity. The greatest decrease was predicted for etravirine, reaching a 50% decrease in AUC_t_ in individuals with a BMI of 50–60 kg/m^2^ (Figure 5). Efavirenz, etravirine, and rilpivirine average C_τ_ concentrations also decreased with increasing BMI. The percentage of individuals below the efficacy target threshold increased, especially for etravirine and rilpivirine, from approximately 10% for a BMI of 18.5–25 kg/m^2^ to approximately 40% for a BMI greater than 40 kg/m^2^. On the other hand, doravirine C_τ_ concentrations were predicted to be unaltered across all obesity classes, which was also supported by the fact that few individuals had predicted doravirine C_τ_ levels below the efficacy threshold (Table 2). The TDM data were mostly available for the lowest obesity category (BMI: 30–35 kg/m^2^) and showed that obese individuals did not have detectable viral load at a higher rate due to lower plasma concentrations, in agreement with our simulations results (Table 3).

#### Integrase Inhibitors

Virtual clinical trials showed that dolutegravir, bictegravir, and raltegravir C_max_ and AUC_t_ were reduced in obese individuals. Bictegravir and raltegravir exposures were predicted to be reduced by more than 25% starting from a BMI greater than 40 kg/m^2^, while for dolutegravir this decrease occurred already in individuals with a BMI greater than 30 kg/m^2^. Nonetheless, for all 3 integrase inhibitors, the percentages of individuals with C_τ_ concentrations below the efficacy threshold were similar across the 6 BMI groups, given that C_τ_ concentrations were minimally affected by obesity compared with AUC_t_ (Figure 5). This finding was further supported by the observation that a viral load greater than 50 copies/mL was not observed more often in obese individuals compared with nonobese individuals (Table 3).

#### Nucleoside/Nucleotide Reverse Transcriptase Inhibitors

With regard to the other ARVs, obesity lowered the exposure of emtricitabine and tenofovir with a reduction in C_max_ and AUC_t_ of more than 25% starting from a BMI greater than 40 kg/m^2^ (Figure 5). Emtricitabine C_τ_ was not altered, whereas tenofovir C_τ_ was reduced by more than 25% starting from a BMI greater than 40 kg/m^2^.

## DISCUSSION

The prevalence of obese PWH has increased in the recent years due to changes in lifestyle [21], diet [22], demographics of the HIV population with a higher proportion of older individuals [23], earlier HIV treatment initiation [2], and growing evidence that several modern ARVs (ie, integrase inhibitors, tenofovir alafenamide) are associated with weight gain [3, 9, 10]. Since obese PWH are underrepresented in clinical trials, the impact of obesity on ARVs exposure and response is incompletely understood. Obesity is associated with physiological changes, which can elevate the metabolic clearance due to an increased cardiac output and consequently increased liver blood flow [5]. Obesity also increases the glomerular filtration rate and thereby can impact the exposure of renally cleared drugs [5]. To date, only 1 study looked at ARVs exposure and response in obese PWH. However, this study did not include contemporary ARVs and did not thoroughly evaluate the impact of various degrees of obesity on the pharmacokinetics [24].

To address this knowledge gap, we combined data of the SHCS on ARVs exposure and response in obese PWH together with PBPK modelling to investigate the full pharmacokinetic profile of 11 contemporary ARVs for different BMI categories.

Rich datasets were available for most ARVs, which allowed us to verify the predictive performance of the PBPK model. Our simulations showed that obesity reduces the exposure of all investigated ARVs. Differences in the magnitude of the decrease in AUC_t_ and C_max_ were found among ARVs, with etravirine showing the highest and doravirine the lowest change. On the other hand, trough concentrations were less impacted by obesity than AUC_t_ and C_max_ because the accumulated drug in the tissues redistributes into the bloodstream, thereby mitigating the effect of obesity on C_τ_. This is important considering that trough concentrations are mostly associated with ARV response. Doravirine C_τ_ was predicted to be even slightly increased at higher BMI, in line with a clinical study showing a 22% increase in doravirine C_τ_ in obese compared with nonobese PWH [25]. Emtricitabine, raltegravir, and darunavir/ritonavir C_τ_ were not significantly decreased with increasing BMI. This finding is also consistent with clinical studies reporting no significant effect of obesity on emtricitabine [24, 26] and darunavir/ritonavir C_τ_ [24]. In contrast to our analysis, Madelain et al [24] reported that raltegravir C_τ_ was significantly lower in obese individuals. However, when plotting their raltegravir trough value in our simulated raltegravir profile (Figure 3E), their value in nonobese PWH is higher compared with our simulations and observed data. Thus, their interpretation could possibly be due to an artefact related to the higher C_τ_ observed in the nonobese group. The remaining evaluated ARVs had to varying extents decreased C_τ_ values at higher BMIs.

Dolutegravir simulations showed a constant decrease in C_τ_ over the studied BMI range, reaching a 34% reduction in the highest BMI group. One clinical trial in nonobese and obese Black African PWH (median BMI: 25.3 vs 32.8 kg/m^2^, respectively) reported a reduction in C_τ_ of 1%, in AUC_t_ of 9%, and in C_max_ of 14% [27], which is in line with our predictions in these BMI groups (Table 1 and Figure 5). These results, together with the fact that the physiology of White and Black obese individuals is similar [28] and that dolutegravir is metabolized by CYP3A4 and UGT1A1, 2 enzymes that are not subject to genetic polymorphism in the Black population, suggest that the results of our simulations for dolutegravir can also be extrapolated to Black PWH.

Clinical studies have reported lower tenofovir C_τ_ in obese PWH, a change that was not considered to be clinically relevant [24, 29]. In our study, tenofovir C_τ_ was also predicted to be lower, particularly in morbidly obese individuals; however, the clinical relevance is unclear considering that the efficacy of nucleoside/nucleotide reverse transcriptase inhibitors relates to the intracellular concentrations. Efavirenz trough concentrations were also predicted to be significantly lower in obese individuals; however, the percentage of virtual individuals below the revisited efficacy target threshold (Table 2) and the virological response (Table 3) were similar between the obese and nonobese groups, consistent with previous observations [24, 26, 30, 31].

Even though obesity caused a reduction in exposure, both predicted and observed trough concentrations, which are linked to ARV efficacy, were less impacted. Importantly, the rate of unsuppressed viral load was not higher in obese compared with nonobese PWH. A few studies have looked at virological responses in obese PWH, most of them were treated with emtricitabine/TDF with efavirenz, darunavir/ritonavir, or raltegravir, and all of them were suppressed with a viral load of less than 50 copies/mL [26, 29, 32, 33]. Available studies and data of the SHCS allowed us to evaluate the effect of obesity up to a BMI of approximately 45 kg/m^2^ (although fewer TDM data were available for etravirine, rilpivirine, doravirine, and tenofovir). Data in morbidly obese PWH (BMI >40 kg/m^2^) are scarce but, based on our analysis, the most impacted ARVs are etravirine and rilpivirine with C_τ_ lower than the clinical target threshold, resulting in potentially suboptimal drug coverage and related risk of treatment failure. Thus, TDM is advised for these ARVs.

The exposure of emtricitabine and TDF was modestly impacted in obese individuals, suggesting that no dose adjustment is required for pre-exposure prophylaxis (PrEP). However, our simulations indicate that tenofovir C_τ_ is reduced by up to 30% in morbidly obese individuals (Figure 5); therefore, PrEP should be used with caution in this particular group.

The strength of this study is the combined use of clinical and modelling data, which allowed us to investigate the full pharmacokinetic profile of oral ARVs for different obesity classes and to evaluate the treatment response. Our methodology can also be applied to study the effect of obesity on long-acting ARVs.

## Conclusions

Obesity lowers the exposure of ARVs; nevertheless, the minimal concentrations of all evaluated ARVs were maintained above the target threshold, except for etravirine and rilpivirine in morbidly obese individuals in whom TDM is advised. When considering the data of the SHCS, the proportion of individuals with viral loads above 50 copies/mL was not higher in obese compared with nonobese PWH. Thus, a dose adjustment of ARVs is a priori not required in obese PWH. Our data provide reassurance that substantial weight gain observed in some individuals on treatment with integrase inhibitors and/or tenofovir alafenamide is unlikely to result in suboptimal drug exposure and response.

## Supplementary Data

Supplementary materials are available at *Clinical Infectious Diseases* online. Consisting of data provided by the authors to benefit the reader, the posted materials are not copyedited and are the sole responsibility of the authors, so questions or comments should be addressed to the corresponding author.

## Supplementary Material

ciad495_Supplementary_Data

## Notes

***Members of the Swiss HIV Cohort Study.*** Irene Abela, Karoline Aebi-Popp, Alexia Anagnostopoulos, Manuel Battegay, Enos Bernasconi, Dominique Laurent Braun, Heiner Bucher, Alexandra Calmy, Matthias Cavassini, Angela Ciuffi, Günter Dollenmaier, Matthias Egger, Luigia Elzi, Jan Fehr, Jacques Fellay, Hansjakob Furrer, Christoph Fux, Huldrych Günthard (president of the Swiss HIV Cohort Study), Anna Hachfeld, David Haerry (deputy of the Positive Council), Barbara Hasse, Hans Hirsch, Matthias Hoffmann, Irene Hösli, Michael Huber, David Jackson-Perry (patient representative), Christian Kahlert (chairman of the Mother and Child Substudy), Laurent Kaiser, Olivia Keiser, Thomas Klimkait, Roger Dimitri Kouyos, Helen Kovari, Katharina Kusejko (head of the data center), Niklaus Labhardt, Karoline Leuzinger, Begona Martinez de Tejada, Catia Marzolini, Karin J. Metzner, Nicolas Müller, Johannes Nemeth, Dunja Nicca, Julia Notter, Paolo Paioni, Giuseppe Pantaleo, Matthieu Perreau, Andri Rauch (chairman of the scientific board), Luisa Salazar-Vizcaya, Patrick Schmid, Roberto Speck, Marcel Stöckle (chairman of the Clinical and Laboratory Committee), Philip Tarr, Alexandra Trkola, Gilles Wandeler, Maja Weisser, and Sabine Yerly.

***Financial support*.** This work was supported by the Swiss National Science Foundation (grant number 188504).
