# Supplementary material for: Antiretroviral Drug Exposure and Response in Obese and Morbidly Obese People With Human Immunodeficiency Virus (HIV): A Study Combining Modelling and Swiss HIV Cohort Data
Source: Clin Infect Dis. 2023 Aug 21;78(1):98–110. doi: 10.1093/cid/ciad495 (PMC10810714; doi:10.1093/cid/ciad495)
Supplement: ciad495_Supplementary_Data [file ciad495_supplementary_data.docx]

Clinical Infectious Diseases

Antiretroviral drug exposure and response in obese and morbidly obese people with HIV: a study combining modelling and Swiss HIV Cohort data

Supplementary Material

Authors: Mattia Berton^1, 2^, Sara Bettonte^1, 2^, Felix Stader^3^, Laurent Decosterd^4^, Philip E. Tarr^5^, Françoise Livio^4^, Matthias Cavassini^6^, Dominique L. Braun^7^, Katharina Kusejko^7^, Anna Hachfeld^8^, Enos Bernasconi^9^, Alexandra Calmy^10^, Patrick Schmid^11^, Manuel Battegay^1, 2^, and Catia Marzolini^1, 2, 4, 12^

Affiliations: 1 Division of Infectious Diseases and Hospital Epidemiology, Departments of Medicine and Clinical Research, University Hospital Basel, 4031 Basel, Switzerland.

2 Faculty of Medicine, University of Basel, 4031 Basel, Switzerland.

3 Certara UK Limited, Sheffield, UK.

4 Service and Laboratory of Clinical Pharmacology, Department of Laboratory Medicine and Pathology, University Hospital Lausanne and University of Lausanne, Switzerland.

5 Kantonsspital Baselland, University of Basel, Bruderholz, Switzerland.

6 Service of Infectious Diseases, Lausanne University Hospital, University of Lausanne, Switzerland.

7 Department of Infectious Diseases and Hospital Epidemiology, University Hospital Zurich, University of Zurich, Switzerland.

8 Department of Infectious Diseases, University Hospital Bern, University of Bern, Switzerland.

9 Division of Infectious Diseases, Ente Ospedaliero Cantonale Lugano, University of Geneva and University of Southern Switzerland, Lugano, Switzerland.

10 Division of Infectious Diseases, University Hospital Geneva, University of Geneva, Switzerland.

11 Department of Infectious Diseases and Hospital Epidemiology, Cantonal Hospital St Gallen, Switzerland.

12 Department of Molecular and Clinical Pharmacology, University of Liverpool, United Kingdom.

Complete contact information for the corresponding authors:

Mattia Berton, MS

Division of Infectious Diseases and Hospital Epidemiology

Departments of Medicine and Clinical Research

University Hospital Basel

Petersgraben 4

4031 Basel, Switzerland

E-mail: mattia.berton@unibas.ch

*Alternative corresponding author:*

Catia Marzolini, PharmD, PhD

Division of Infectious Diseases and Hospital Epidemiology

Departments of Medicine and Clinical Research

University Hospital Basel

Petersgraben 4

4031 Basel, Switzerland

E-mail: catia.marzolini@usb.ch

Swiss HIV Cohort Study

The Swiss HIV Cohort Study (SHCS) is an ongoing, nationwide, multicenter, clinic-based prospective cohort study in Switzerland with continuous enrollment and follow-up (twice a year) of people with HIV (PWH). Socio-demographic, clinical and laboratory data are recorded in detail. The body mass index (BMI) distribution in 2022 considering all active SHCS participants (n = 9471) was: underweight (BMI <18.5 kg/m^2^) n = 316 (3.3%); normal weight (BMI 18.5-24.9 kg/m^2^) n = 4284 (45.2%); overweight (BMI 25-29.9 kg/m^2^) n = 3389 (35.8%); obesity category I (BMI 30-34.9 kg/m^2^) n = 1128 (11.9%); obesity category class II (BMI 35-39.9 kg/m^2^) n= 264 (2.8%) and obesity category III and over (BMI >40 kg/m^2^) 90 (1%). The collection of therapeutic drug monitoring (TDM) data in the SHCS database was initiated in 1998. All available TDM data for each evaluated antiretroviral drug were extracted from the SHCS database representing 6210 and 575 data points for the verification of the simulations in non-obese and obese individuals, respectively.

Supplementary Table 1. Doravirine PBPK model parameters.

| **Parameter** | **Unit** | **Doravirine** | **Reference** |
| --- | --- | --- | --- |
| molecular weight | g/mol | 452.7 | [1] |
| log P |  | 3.0 | [1] |
| drug type |  | Monoprotic base | [1] |
| pK_a1_ |  | 9.5 | [1] |
| BP |  | 1.0 | [1] |
| fu_p_ |  | 0.24 | [1] |
| Apparent permeability | 10^-6^ cm/sec | 25 | [1] |
| CYP3A4 CL_int_ | µl/min/pmol enzyme | 0.032 | Retrograde calculation |
| CL_renal_ | L/h | 0.566 | [1] |

**Abbreviations:** BP, blood-plasma-ratio; CL_int_, intrinsic clearance; CL_renal_, renal clearance; CYP, cytochrome P-450; fu_p_, fraction unbound in plasma; log P, octanol-water partition coefficient; pk_a_, acid dissociation constant.

Supplementary Table 2. Summary of study design and demographic parameters mean (range) for the different antiretroviral drugs investigated in non-obese and obese people with HIV.

| **Drug** | **Dosing** | **Non-obese individuals** | | | | | **Obese individuals** | | | | |
| --- | --- | --- | --- | --- | --- | --- | --- | --- | --- | --- | --- |
|  | **regimen** |  |  |  |  |  |  |  |  |  |  |
|  |  | Reference | Number of individuals | Age  [years] | Proportion  female | BMI  [kg/m²] | Reference | Number of individuals | Age  [years] | Proportion  female | BMI  [kg/m²] |
| Ritonavir | 100mg QD, PO, S.S. | [2-5] | 156 | 39 (20-49) | 0.31 | 24 (19-30) | [4, 5] | 55 | 41 (24-50) | 0.61 | 35 (30-51) |
| Darunavir / ritonavir | 800mg/100mg QD, PO, S.S. | [3-7] | 103 | 44 (20-53) | 0.46 | 24 (20-30) | [4, 5] | 49 | 40 (22-50) | 0.53 | 34 (30-42) |
| Efavirenz | 600mg QD, PO, S.S. | [4, 5, 8] | 2588 | 39 (20-50) | 0.26 | 23 (19-30) | [4, 5] | 218 | 41 (21-50) | 0.55 | 33 (30-60) |
| Etravirine | 200mg BID, PO, S.S. | [9-11] | 75 | 42 (24-54) | 0.4 | 25 (20-31) | [5] | 35 | 43 (34-49) | 0.69 | 36 (30-51) |
| Rilpivirine | 25mg QD, PO, S.S. | [12-14] | 66 | 30 (20-46) | 0.26 | 25 (22-30) | [5] | 16 | 44 (28-50) | 0.44 | 33 (30-38) |
| Doravirine | 100mg QD, PO, S.S. | [15, 16] | 26 | 39 (24-56) | 0.23 | 27 (20-30) | [5, 16] | 14 | 50 (32-56) | 0.72 | 0 (30-41) |
| Dolutegravir | 50mg, PO, S.S. | [5, 14, 17, 18] | 524 | 39 (20-51) | 0.28 | 24 (19-30) | [5] | 56 | 42 (20-50) | 0.55 | 33 (30-42) |
| Bictegravir | 50mg, PO, S.S. | [5] | 122 | 39 (23-50) | 0.34 | 25 (19-30) | [5] | 26 | 42 (24-50) | 0.81 | 34 (30-44) |
| Raltegravir | 400mg, BID, S.S. | [4, 5, 19, 20] | 233 | 41 (21-50) | 0.3 | 24 (19-30) | [4, 5] | 50 | 44 (29-50) | 0.52 | 35 (30-52) |
| Emtricitabine | 200mg QD, PO, S.S. | [13, 21-23] | 60 | 29 (23-49) | 0.49 | 24 (21-33) | [5] | 13 | 43 (24-50) | 0.86 | 35 (30-46) |
| Tenofovir Disoproxil Fumarate | 300mg QD, PO, S.S. non-obese; 245mg QD, PO, S.S. obese; | [21, 22, 24] | 44 | 31 (20-51) | 0.33 | 24 (20-30) | [25] | 4 | 43 (40-46) | 0.75 | 43 (36-46) |

BID, twice daily, BMI, body mass index, PO, oral administration route, QD, once daily, S.S., steady state

Supplementary Table 3. Simulated changes in the pharmacokinetic parameters across different BMI classes.

|  |  | C_max_ (ng/mL) | C_max_ (ng/mL) | C_max_ (ng/mL) | AUC_t_ (ng*h/mL) | AUC_t_ (ng*h/mL) | AUC_t_ (ng*h/mL) | C_τ_ (ng/mL) | C_τ_ (ng/mL) | C_τ_ (ng/mL) |
| --- | --- | --- | --- | --- | --- | --- | --- | --- | --- | --- |
| Drug | BMI (kg/m^2^) | Geomean | CI 5 | CI 95 | Geomean | CI 5 | CI 95 | Geomean | CI 5 | CI 95 |
| Ritonavir | 18.5-25 | 633 | 305 | 1624 | 7783 | 2870 | 29221 | 94 | 20 | 763 |
| Ritonavir | 25-30 | 530 | 238 | 1448 | 6779 | 2763 | 24961 | 87 | 23 | 641 |
| Ritonavir | 30-35 | 509 | 284 | 976 | 5596 | 2657 | 15175 | 51 | 12 | 325 |
| Ritonavir | 35-40 | 397 | 231 | 888 | 5093 | 2475 | 15297 | 68 | 20 | 376 |
| Ritonavir | 40-50 | 412 | 247 | 877 | 5162 | 2452 | 15678 | 67 | 14 | 397 |
| Ritonavir | 50-60 | 316 | 172 | 771 | 4995 | 2456 | 14704 | 103 | 29 | 441 |
|  |  |  |  |  |  |  |  |  |  |  |
| Darunavir | 18.5-25 | 6405 | 3818 | 11274 | 92444 | 45853 | 201993 | 1568 | 396 | 5399 |
| Darunavir | 25-30 | 5896 | 3971 | 10684 | 86740 | 49613 | 190319 | 1775 | 774 | 5634 |
| Darunavir | 30-35 | 5330 | 3386 | 9616 | 79079 | 43507 | 176973 | 1716 | 763 | 5311 |
| Darunavir | 35-40 | 4757 | 3213 | 8579 | 71028 | 43628 | 157492 | 1603 | 785 | 4707 |
| Darunavir | 40-50 | 4216 | 2671 | 6772 | 64913 | 37296 | 118405 | 1555 | 708 | 3585 |
| Darunavir | 50-60 | 3979 | 2665 | 6886 | 64267 | 40608 | 133936 | 1684 | 878 | 4513 |
|  |  |  |  |  |  |  |  |  |  |  |
| Efavirenz | 18.5-25 | 4267 | 2926 | 6605 | 59079 | 32721 | 110563 | 1889 | 905 | 4159 |
| Efavirenz | 25-30 | 3716 | 2316 | 6090 | 49323 | 25850 | 96329 | 1602 | 745 | 3591 |
| Efavirenz | 30-35 | 3515 | 2215 | 6244 | 46376 | 24944 | 104415 | 1537 | 723 | 3974 |
| Efavirenz | 35-40 | 3426 | 2296 | 5110 | 45583 | 27116 | 81338 | 1552 | 832 | 3106 |
| Efavirenz | 40-50 | 3071 | 2178 | 4509 | 40513 | 23953 | 70242 | 1401 | 757 | 2654 |
| Efavirenz | 50-60 | 2709 | 1824 | 4199 | 34903 | 20066 | 61179 | 1216 | 630 | 2288 |
|  |  |  |  |  |  |  |  |  |  |  |
| Etravirine | 18.5-25 | 935 | 485 | 1658 | 7855 | 3616 | 15774 | 477 | 185 | 1145 |
| Etravirine | 25-30 | 799 | 491 | 1580 | 6450 | 3573 | 14078 | 384 | 187 | 982 |
| Etravirine | 30-35 | 733 | 383 | 1345 | 5874 | 2852 | 12587 | 351 | 146 | 858 |
| Etravirine | 35-40 | 622 | 398 | 1081 | 4933 | 2804 | 9370 | 293 | 153 | 621 |
| Etravirine | 40-50 | 580 | 321 | 987 | 4445 | 2044 | 8080 | 259 | 102 | 552 |
| Etravirine | 50-60 | 495 | 295 | 905 | 3708 | 2022 | 7488 | 216 | 102 | 508 |
|  |  |  |  |  |  |  |  |  |  |  |
| Rilpivirine | 18.5-25 | 173 | 118 | 258 | 2903 | 1929 | 4705 | 84 | 52 | 149 |
| Rilpivirine | 25-30 | 150 | 109 | 222 | 2453 | 1755 | 3763 | 72 | 49 | 119 |
| Rilpivirine | 30-35 | 143 | 109 | 190 | 2314 | 1752 | 3409 | 69 | 50 | 113 |
| Rilpivirine | 35-40 | 132 | 97 | 196 | 2155 | 1529 | 3337 | 66 | 44 | 109 |
| Rilpivirine | 40-50 | 121 | 93 | 181 | 1945 | 1474 | 3151 | 60 | 44 | 101 |
| Rilpivirine | 50-60 | 107 | 82 | 150 | 1704 | 1274 | 2468 | 53 | 39 | 82 |
|  |  |  |  |  |  |  |  |  |  |  |
| Doravirine | 18.5-25 | 1371 | 953 | 1862 | 16905 | 11493 | 23887 | 296 | 170 | 477 |
| Doravirine | 25-30 | 1220 | 876 | 1855 | 15525 | 10715 | 24237 | 314 | 200 | 547 |
| Doravirine | 30-35 | 1132 | 772 | 1554 | 14887 | 10504 | 20941 | 325 | 223 | 498 |
| Doravirine | 35-40 | 1030 | 752 | 1441 | 14239 | 10404 | 20212 | 335 | 241 | 519 |
| Doravirine | 40-50 | 956 | 627 | 1457 | 13107 | 8600 | 19259 | 318 | 210 | 473 |
| Doravirine | 50-60 | 838 | 558 | 1276 | 12084 | 8270 | 18395 | 314 | 204 | 500 |
|  |  |  |  |  |  |  |  |  |  |  |
| Dolutegravir | 18.5-25 | 3617 | 2313 | 5564 | 51499 | 25226 | 99359 | 945 | 233 | 2823 |
| Dolutegravir | 25-30 | 2955 | 2110 | 4628 | 42452 | 22887 | 84581 | 810 | 220 | 2563 |
| Dolutegravir | 30-35 | 2571 | 1620 | 4436 | 37945 | 17831 | 84040 | 765 | 182 | 2583 |
| Dolutegravir | 35-40 | 2314 | 1459 | 4063 | 34813 | 14809 | 79633 | 729 | 160 | 2670 |
| Dolutegravir | 40-50 | 2002 | 1306 | 3526 | 30931 | 15334 | 69700 | 690 | 186 | 2262 |
| Dolutegravir | 50-60 | 1693 | 1133 | 2555 | 26761 | 14108 | 49048 | 625 | 203 | 1573 |
|  |  |  |  |  |  |  |  |  |  |  |
| Bictegravir | 18.5-25 | 4647 | 2539 | 8655 | 80191 | 34528 | 178057 | 2151 | 581 | 6082 |
| Bictegravir | 25-30 | 4023 | 2478 | 7547 | 72136 | 38979 | 160710 | 2083 | 834 | 5759 |
| Bictegravir | 30-35 | 3935 | 2147 | 7827 | 73326 | 34015 | 172501 | 2251 | 826 | 6347 |
| Bictegravir | 35-40 | 3681 | 2156 | 7286 | 68171 | 34199 | 154945 | 2128 | 809 | 5737 |
| Bictegravir | 40-50 | 3195 | 1812 | 6691 | 59519 | 26639 | 146905 | 1889 | 683 | 5659 |
| Bictegravir | 50-60 | 2704 | 1605 | 6239 | 50715 | 26603 | 137073 | 1634 | 704 | 5303 |
|  |  |  |  |  |  |  |  |  |  |  |
| Raltegravir | 18.5-25 | 2224 | 1556 | 3525 | 7756 | 5208 | 14594 | 121 | 60 | 333 |
| Raltegravir | 25-30 | 1990 | 1449 | 2666 | 7084 | 5264 | 10156 | 134 | 79 | 236 |
| Raltegravir | 30-35 | 1873 | 1280 | 2937 | 6618 | 4861 | 11034 | 132 | 83 | 287 |
| Raltegravir | 35-40 | 1678 | 1202 | 2685 | 6227 | 4338 | 10762 | 135 | 82 | 279 |
| Raltegravir | 40-50 | 1442 | 1026 | 2029 | 5252 | 3782 | 7298 | 118 | 74 | 189 |
| Raltegravir | 50-60 | 1280 | 891 | 2004 | 4665 | 3294 | 8108 | 113 | 74 | 267 |
|  |  |  |  |  |  |  |  |  |  |  |
| Emtricitabine | 18.5-25 | 1806 | 1516 | 2156 | 11791 | 10065 | 14290 | 91 | 53 | 141 |
| Emtricitabine | 25-30 | 1653 | 1378 | 2002 | 11092 | 9241 | 13577 | 108 | 76 | 155 |
| Emtricitabine | 30-35 | 1548 | 1291 | 1888 | 10610 | 8924 | 13030 | 114 | 80 | 166 |
| Emtricitabine | 35-40 | 1330 | 1104 | 1596 | 8882 | 7465 | 10604 | 93 | 68 | 133 |
| Emtricitabine | 40-50 | 1309 | 1073 | 1603 | 9407 | 7742 | 11570 | 119 | 87 | 175 |
| Emtricitabine | 50-60 | 1115 | 915 | 1344 | 8091 | 6899 | 9668 | 108 | 79 | 153 |
|  |  |  |  |  |  |  |  |  |  |  |
| Tenofovir | 18.5-25 | 337 | 264 | 429 | 3672 | 2833 | 4777 | 80 | 55 | 119 |
| Tenofovir | 25-30 | 314 | 251 | 402 | 3360 | 2609 | 4400 | 77 | 53 | 111 |
| Tenofovir | 30-35 | 283 | 227 | 353 | 2899 | 2326 | 3609 | 66 | 48 | 89 |
| Tenofovir | 35-40 | 282 | 225 | 360 | 3016 | 2348 | 3922 | 73 | 52 | 102 |
| Tenofovir | 40-50 | 237 | 188 | 298 | 2309 | 1854 | 2819 | 53 | 39 | 70 |
| Tenofovir | 50-60 | 226 | 181 | 277 | 2319 | 1853 | 2887 | 57 | 43 | 76 |

AUC_t_, area under the curve to time t, BMI, body mass index, CI 5, confidence interval 5^th^ percentile, CI 95, confidence interval 95^th^ percentile, C_max_, peak concentration, C_τ,_ trough concentration

**References**

1. Yee KL, Cabalu TD, Kuo Y, et al. Physiologically based pharmacokinetic modeling of doravirine and Its major metabolite to support dose adjustment with rifabutin. J Clin Pharmacol **2021**; 61(3): 394-405.

2. Mathias AA, West S, Hui J, Kearney BP. Dose-response of ritonavir on hepatic CYP3A activity and elvitegravir oral exposure. Clin Pharmacol Ther **2009**; 85(1): 64-70.

3. DeJesus E, Lalezari JP, Osiyemi OO, et al. Pharmacokinetics of once-daily etravirine without and with once-daily darunavir/ritonavir in antiretroviral-naive HIV type-1-infected adults. Antivir Ther **2010**; 15(5): 711-20.

4. Madelain V, Le MP, Champenois K, et al. Impact of obesity on antiretroviral pharmacokinetics and immuno-virological response in HIV-infected patients: a case-control study. J Antimicrob Chemother **2017**; 72(4): 1137-46.

5. Swiss HIV Cohort Study, Therapeutic drug monitoring data. www.shcs.ch.

6. Boffito M, Miralles D, Hill A. Pharmacokinetics, efficacy, and safety of darunavir/ritonavir 800/100 mg once-daily in treatment-naive and -experienced patients. HIV Clin Trials **2008**; 9(6): 418-27.

7. Kakuda TN, Opsomer M, Timmers M, et al. Pharmacokinetics of darunavir in fixed-dose combination with cobicistat compared with coadministration of darunavir and ritonavir as single agents in healthy volunteers. J Clin Pharmacol **2014**; 54(8): 949-57.

8. Villani P, Regazzi MB, Castelli F, et al. Pharmacokinetics of efavirenz (EFV) alone and in combination therapy with nelfinavir (NFV) in HIV-1 infected patients. Br J Clin Pharmacol **1999**; 48(5): 712-5.

9. Scholler-Gyure M, Kakuda TN, De Smedt G, et al. Pharmacokinetics of TMC125 in once- and twice-daily regimens in HIV-1-negative volunteers. 47th Interscience Conference on Antimicrobial Agents and Chemotherapy. Chicago, IL, USA, **2007**.

10. Scholler-Gyure M, Kakuda TN, De Smedt G, et al. Effects of hepatic impairment on the steady-state pharmacokinetics of etravirine 200 mg BID: an open-label, multiple-dose, controlled Phase I study in adults. Clin Ther **2010**; 32(2): 328-37.

11. Anderson MS, Kakuda TN, Hanley W, et al. Minimal pharmacokinetic interaction between the human immunodeficiency virus nonnucleoside reverse transcriptase inhibitor etravirine and the integrase inhibitor raltegravir in healthy subjects. Antimicrob Agents Chemother **2008**; 52(12): 4228-32.

12. Crauwels H, Vingerhoets J, Ryan R, Witek J, Anderson D. Pharmacokinetic parameters of once-daily rilpivirine following administration of efavirenz in healthy subjects. Antivir Ther **2012**; 17(3): 439-46.

13. Dickinson L, Yapa HM, Jackson A, et al. Plasma tenofovir, emtricitabine, and rilpivirine and intracellular tenofovir diphosphate and emtricitabine triphosphate pharmacokinetics following drug intake cessation. Antimicrob Agents Chemother **2015**; 59(10): 6080-6.

14. Ford SL, Gould E, Chen S, et al. Lack of pharmacokinetic interaction between rilpivirine and integrase inhibitors dolutegravir and GSK1265744. Antimicrob Agents Chemother **2013**; 57(11): 5472-7.

15. Yee KL, Sanchez RI, Auger P, et al. Evaluation of doravirine pharmacokinetics when switching from efavirenz to doravirine in healthy subjects. Antimicrob Agents Chemother **2017**; 61(2).

16. Zino L. et al. Doravirine exposure in obese population living with HIV infection (Double Study): data from physiologically-based pharmacokinetics modelling and real-life patients. International Workshop on Clinical Pharmacology. Barcelona, Spain, 19-20 September **2022**.

17. Elliot ER, Wang X, Singh S, et al. Increased dolutegravir peak concentrations in people living with human immunodeficiency virus aged 60 and over, and analysis of sleep quality and cognition. Clin Infect Dis **2019**; 68(1): 87-95.

18. Song I, Borland J, Min S, et al. Effects of etravirine alone and with ritonavir-boosted protease inhibitors on the pharmacokinetics of dolutegravir. Antimicrob Agents Chemother **2011**; 55(7): 3517-21.

19. Iwamoto M, Wenning LA, Petry AS, et al. Minimal effects of ritonavir and efavirenz on the pharmacokinetics of raltegravir. Antimicrob Agents Chemother **2008**; 52(12): 4338-43.

20. Markowitz M, Morales-Ramirez JO, Nguyen BY, et al. Antiretroviral activity, pharmacokinetics, and tolerability of MK-0518, a novel inhibitor of HIV-1 integrase, dosed as monotherapy for 10 days in treatment-naive HIV-1-infected individuals. J Acquir Immune Defic Syndr **2006**; 43(5): 509-15.

21. Blum MR, Chittick GE, Begley JA, Zong J. Steady-state pharmacokinetics of emtricitabine and tenofovir disoproxil fumarate administered alone and in combination in healthy volunteers. J Clin Pharmacol **2007**; 47(6): 751-9.

22. Ramanathan S, Shen G, Cheng A, Kearney BP. Pharmacokinetics of emtricitabine, tenofovir, and GS-9137 following coadministration of emtricitabine/tenofovir disoproxil fumarate and ritonavir-boosted GS-9137. J Acquir Immune Defic Syndr **2007**; 45(3): 274-9.

23. Wang LH, Begley J, St Claire RL, 3rd, Harris J, Wakeford C, Rousseau FS. Pharmacokinetic and pharmacodynamic characteristics of emtricitabine support its once daily dosing for the treatment of HIV infection. AIDS Res Hum Retroviruses **2004**; 20(11): 1173-82.

24. Kearney BP, Mathias A, Mittan A, Sayre J, Ebrahimi R, Cheng AK. Pharmacokinetics and safety of tenofovir disoproxil fumarate on coadministration with lopinavir/ritonavir. JAIDS Journal of Acquired Immune Deficiency Syndromes **2006**; 43(3): 278-83.

25. Muzard L, Alvarez JC, Gbedo C, Czernichow S, Carette C. Tenofovir pharmacokinetic after sleeve-gastrectomy in four severely obese patients living with HIV. Obes Res Clin Pract **2017**; 11(1): 108-13.
